# Grade Classification of Camellia Seed Oil Based on Hyperspectral Imaging Technology

**DOI:** 10.3390/foods13203331

**Published:** 2024-10-20

**Authors:** Yuqi Gu, Jianhua Wu, Yijun Guo, Sheng Hu, Kaixuan Li, Yuqian Shang, Liwei Bao, Muhammad Hassan, Chao Zhao

**Affiliations:** 1 College of Optical, Mechanical and Electrical Engineering, Zhejiang A&F University, Hangzhou 311300, China; guyuqi@zafu.edu.cn (Y.G.); pop15779972603@outlook.com (Y.G.); hs15057087528@163.com (S.H.); baoliwei@stu.zafu.edu.cn (L.B.); 2 Panzhihua Academy of Agriculture and Forestry Sciences, Panzhihua 617061, China; jhuawu2024@163.com; 3 National Engineering Technology Research Center of Forestry and Grassland Machinery for Hilly and Mountainous Areas, State Forestry and Grassland Administration, Hangzhou 311300, China; 2023612021010@stu.zafu.edu.cn; 4 Key Laboratory of Agricultural Equipment for Hilly and Mountainous Areas in Southeastern China, Ministry of Agriculture and Rural Affairs, Hangzhou 311300, China; shangyuqian@stu.zafu.edu.cn; 5 U.S.-Pakistan Center for Advanced Studies in Energy, National University of Sciences and Technology (NUST), Islamabad 44000, Pakistan; hassan@uspcase.nust.edu.pk

**Keywords:** camellia seed oil, grade classification, hyperspectral imaging technology, characteristic wavelength

## Abstract

To achieve the rapid grade classification of camellia seed oil, hyperspectral imaging technology was used to acquire hyperspectral images of three distinct grades of camellia seed oil. The spectral and image information collected by the hyperspectral imaging technology was preprocessed by different methods. The characteristic wavelength selection in this study included the continuous projections algorithm (SPA) and competitive adaptive reweighted sampling (CARS), and the gray-level co-occurrence matrix (GLCM) algorithm was used to extract the texture features of camellia seed oil at the characteristic wavelength. Combined with genetic algorithm (GA) and support vector machine algorithm (SVM), different grade classification models for camellia seed oil were developed using full wavelengths (GA-SVM), characteristic wavelengths (CARS-GA-SVM), and fusing spectral and image features (CARS-GLCM-GA-SVM). The results show that the CARS-GLCM-GA-SVM model, which combined spectral and image information, had the best classification effect, and the accuracy of the calibration set and prediction set of the CARS-GLCM-GA-SVM model were 98.30% and 96.61%, respectively. Compared with the CARS-GA-SVM model, the accuracy of the calibration set and prediction set were improved by 10.75% and 12.04%, respectively. Compared with the GA-SVM model, the accuracy of the calibration set and prediction set were improved by 18.28% and 18.15%, respectively. The research showed that hyperspectral imaging technology can rapidly classify camellia seed oil grades.

## 1. Introduction

Camellia seed oil is a vegetable oil extracted from mature fruits picked from camellia trees [1]. Camellia seed oil is rich in polyunsaturated fatty acids such as linolenic acid, linoleic acid, a small amount of saturated fatty acids, and many functional components. It has obvious effects in the cardiovascular system and cerebrovascular system, relieving diabetes, preventing and treating hypertension, improving human immunity, and lowering cholesterol [2]. Because of these, the price of camellia seed oil is much higher than other edible vegetable oils, and there is a high risk of adulteration with low-quality or low-priced vegetable oils [3]. The quality and safety of food are very important and related to human health and development [3,4]. Adulterated or low-quality camellia seed oil not only violates food safety regulations but also poses a serious threat to the consumer’s health. Adulterated or low-quality camellia seed oil destroys market order, damages the rights and interests of regular producers and consumers, and affects the healthy development of the entire industry. Therefore, quickly detecting the quality and grade of camellia seed oil is an urgent issue in the camellia seed oil industry.

At present, various advanced detection technologies have been used in the detection of food and edible oil, such as gas chromatography [5], near infrared spectroscopy [6,7], Raman spectroscopy [8], laser-induced breakdown spectroscopy [9], and hyperspectral imaging technology [10,11]. Generally, these detection methods need to be combined with chemical statistics, multiple linear regression, artificial neural networks, pattern recognition, principal component analysis (PCA), partial least squares regression (PLSR), and linear discriminant analysis (LDA) methods [9,12,13,14]. Spectral information is usually combined with stoichiometry and artificial intelligence to classify edible oils, determine their authenticity qualitatively, or detect their quality or adulteration level quantitatively [2,10]. Regarding the detection of camellia seed oil, He et al. (2018) established a partial least squares regression model of fatty acids in camellia seed oil by near infrared technology, and the correlation coefficients of its calibration set and prediction set were 0.87 and 0.82, respectively [15]. He et al. (2018) further compared the fatty acid content of camellia seed oil in different regions of Jiangxi (102 samples from 36 regions were divided into 4 regions according to geographical latitude) and found that the content of heptadecanoic acid and stearic acid in camellia seed oil increased with the increase in geographical latitude, while the content of unsaturated fatty acids decreased with the increase in geographical latitude [5]. Therefore, the quality and grade of camellia seed oil in different regions are different. Wen et al. (2015) used visible/near infrared technology combined with the competitive adaptive reweighed sampling (CARS) method to detect adulteration of soybean oil and rapeseed oil in camellia seed oil. The research finding indicated that the correlation coefficient of the model of the adulteration level of soybean oil, rapeseed oil, and mixed were 0.950, 0.928, and 0.980, respectively. The CARS method was an effective characteristic wavelength selection method, which could eliminate redundant wavelength variables [16].

As a new green detection technology, hyperspectral imaging technology has the advantages of fast detection speed, no damage to samples, and high accuracy of detection for the quality, adulteration, and grade classification of edible oil [17]. Many studies have applied hyperspectral imaging technology to the detection of edible oil [2,10,18]. Combined with hyperspectral imaging technology and portable infrared technology, Da et al. (2022) realized the identification of Brassicas seed oil varieties and components. The results indicated that the PLS-DA model achieved 100% accuracy in identifying varieties of Brassica seed oil. At the same time, the contents of erucic acid, MUFAs (monounsaturated fatty acids), and PUFAs (polyunsaturated fatty acids) in Brassicas seed oil were quantitatively predicted [19]. Two recent studies have realized the identification of authenticity discrimination and adulteration level detection of camellia seed oil [1,2]. Based on hyperspectral imaging technology, characteristic wavelengths (400~570 nm, 695 nm) were selected, and the classification of olive oil with different flavors was realized [10]. Combined with hyperspectral imaging technology and chemometrics, the oil content of peanuts was predicted accurately. The research concluded that the PLSR model established according to the characteristic wavelengths had the best effect, with a correlation coefficient of the prediction set of 0.934 and a root mean square error of the prediction set (RMSEP) of 0.197%. Hyperspectral imaging technology held great promise as a rapid and nondestructive method for determining peanut oil content [18]. Combined with near infrared hyperspectral imaging technology and a deep convolution network, the oil content of corn seeds was predicted. The research showed that the results predicted by PLSR and SVR models were very close to the experimental results [20].

According to the research mentioned above, many researchers have applied hyperspectral imaging technology to assess the quality of various foods; however, there are few studies on the grade classification of camellia seed oil via hyperspectral imaging technology. At present, the conventional methods for evaluating the grade of edible oil mainly rely on sensory evaluation. This method is not suitable for the detection of large numbers of samples, and it is easily influenced by the appraiser, which leads to a lack of rigor and scientificity of the judgment results.

In this paper, hyperspectral imaging technology was employed to classify the grade of camellia seed oil. The spectral and image information of three different grades of camellia seed oil were collected by hyperspectral imaging technology. Grade classification models for camellia seed oil were developed using genetic algorithms and support vector machines. Different processing methods, characteristic wavelength selection, and image texture information extraction were applied to improve the accuracy of the grade classification model. The feasibility of hyperspectral imaging technology for the grade classification of camellia oil was discussed.

## 2. Materials and Methods

### 2.1. Sample Collection

Three grades of camellia seed oil were used in the experiments, of which Grade 1 was produced by Zhejiang Qiandaohu Food Co., Ltd. (Hangzhou, China), Grade 2 was produced by Jiangxi Qiyunshan Food Co., Ltd. (Ganzhou, China), and Grade 3 was produced by Guangxi Sanjiang Food Co., Ltd. (Guangxi, China). The appearances of the different camellia seed oil grades are shown in Figure S1. The grade classifications of camellia oil were divided according to GB/T 11765-2018 “Oil-tea camellia seed oil” [21]. The quality criteria of the three grades of camellia seed oil are shown in Table 1. A total of 56 samples were collected from each grade, for a total of 168 samples. All samples were stored in a refrigerator at 4 °C until analysis.

### 2.2. Hyperspectral Imaging System and Data Acquisition

#### 2.2.1. Hyperspectral Imaging System

**Figure 1:**
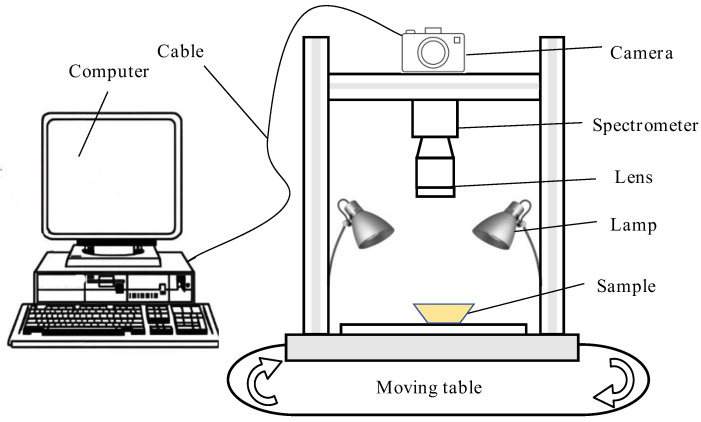
The diagram of the hyperspectral imaging system.

The hyperspectral imaging system used in this study is shown in Figure 1. The system was mainly composed of a mobile workbench (moving samples), four 150 W tungsten light sources (providing a bright environment), a 270 × 310 pixel camera (shooting samples), and a computer and a monitor (for collecting and recording information). After focusing, the shooting parameters of the hyperspectral imaging system were as follows: object distance of 400 mm, light source steady flow of 5.0 A, scanning speed of 150 μm/s, line scanning length of 70 mm, exposure time of the imaging spectrometer of 25 ms, and a spectral resolution of the finally obtained spectral image of 300 pix × 310 pix × 248 pix.

#### 2.2.2. Hyperspectral Data Acquisition and Information Extraction

**Figure 2:**
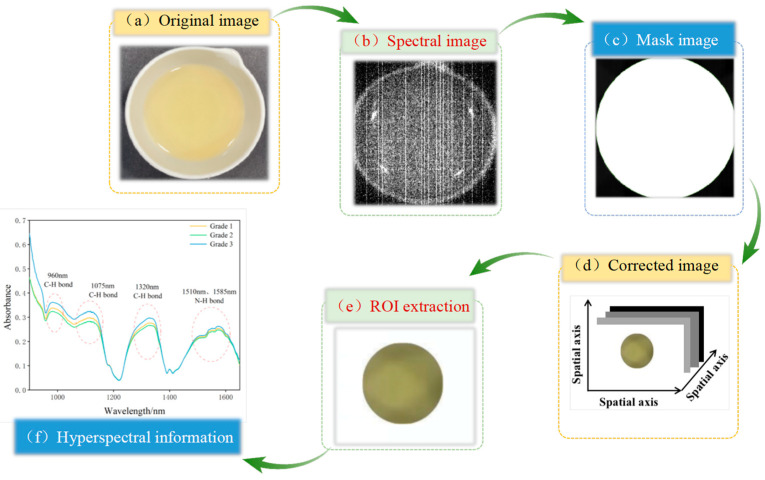
The process of hyperspectral information extraction.

The hyperspectral data collection process for camellia seed oil involved pouring 10 ± 0.05 g of the sample into a 30 mL ceramic evaporating dish. The dish was then placed flat on a moving table and moved at a speed of 25 mm/s. The Spec View software (SpecVIEW v2.9) was used to conduct hyperspectral data acquisition and reflectivity calibration. Six samples were scanned per picture, with each group scanned three times, and the average value was taken for analysis. The process of spectral information extraction is shown in Figure 2. According to the previous studies, the ENVI spectral image processing software was applied to process the collected images, including cropping, background segmentation, and region of interest (ROI) extraction. When extracting the ROI, the camellia seed oil region in each image should be selected and cropped, and the average spectral reflectance of the ROI calculated to serve as the sample’s spectral data.

The abnormal samples were removed during the hyperspectral data collection and processing. Abnormal samples refer to samples whose spectral curves significantly deviate from the normal sample distribution due to operational errors, fluctuations in lighting conditions, or equipment malfunctions during the spectral acquisition process. The abnormal samples were determined based on the Euclidean distance between the standardized spectral curve and other samples exceeding a preset threshold. The removal of abnormal samples could greatly improve the accuracy of modeling. At last, 8 abnormal samples were eliminated from Grade 1 samples and 7 abnormal samples from Grade 2 samples. Therefore, there were 48, 49, and 56 samples of grades 1, 2, and 3 camellia seed oil for subsequent experiments. The total number of samples was 153.

#### 2.2.3. Sample Division

The K-S classification was used to divide the samples. The K-S classification method divides the samples into a calibration set and a prediction set according to the Euclidean distance of the samples in the spectral interval. The samples with the larger Euclidean distance were classified as the calibration set, while the remaining samples were designated as the prediction set. Compared with random classification, the K-S classification could effectively improve the uniformity of sample distribution [22]. According to the ratio of 3:2, the camellia seed oil samples were divided into a calibration set and a prediction set. The results of the sample division are shown in Table 2, in which there were 92 samples in the calibration set and 61 samples in the prediction set.

### 2.3. Spectral Data Preprocessing

Apart from the required sample information, there was a plethora of irrelevant information present in the acquired spectral data, including undercurrent, stray light, background color, and mechanical noise, all of which had a certain impact on the accuracy and stability of the experimental results. To enhance the accuracy and stability of the models and minimize the impact of external interference, the obtained spectral data were preprocessed [23,24]. The preprocessing methods applied in this research included Savitzky Golay smoothing (SG), Normalize, Standard Normal Variate (SNV), First Derivative (1Der), and Second Derivative (2Der). A suitable preprocessing method was screened to improve the accuracy of the model.

### 2.4. Grade Classification Analysis of Camellia Seed Oil

#### 2.4.1. Principal Component Analysis (PCA)

Principal component analysis (PCA) is a method to reduce the dimension of the target data by orthogonal transformation. PCA calculates the linear projection of the original high-dimensional spectral data, transforms these data into a number of unrelated variables, which are called principal components (PCs), and realizes the transformation of data from high dimension to low dimension [24]. In this study, the clustering of different grades of camellia seed oil was analyzed by PCA, and the feasibility of the grade classification of camellia seed oil was investigated. The PCA of this study was completed by the Unscromber X software (The Unscrambler X 11).

#### 2.4.2. Characteristic Wavelength Selection

Full-band spectral data obtained by a hyperspectral imaging system has high dimensions and redundant information, so it takes a lot of calculation and long operation time to establish the model. In order to improve the accuracy and stability of the established model, the characteristic wavelengths were selected during the modeling process. The characteristic wavelength selection in this study included a continuous projections algorithm (SPA) and competitive adaptive reweighted sampling (CARS) [17].

(1) Continuous Projections Algorithm (SPA)

The SPA method searches the variable groups with minimum redundant information in spectral information. It minimizes the influence of collinearity between variable groups by using the projection analysis of vectors, thus reducing the overlap of information. At the same time, several characteristic wavelengths are selected to represent most of the information of the original data, which improves the speed and efficiency of the modeling.

(2) Competitive Adaptive Reweighted Sampling (CARS)

The basic idea of CARS is to optimize the partial least squares regression coefficient according to adaptive reweighting so that the wavelengths with a larger weight distribution are selected, while the wavelengths with a smaller weight distribution are removed. Finally, the dataset with the smallest cross-calibration root mean square error (RMSECV) is selected by the cross-calibration method. Therefore, the characteristic wavelengths with the highest correlation with the label value are selected through the above screening principle.

#### 2.4.3. Image Feature Analysis

Hyperspectral imaging technology could obtain spectral and image information at any wavelength. Color is an important classification index for camellia seed oil according to grading standards (Table 1), and camellia seed oil with different colors presents different features in the image. In order to make full use of the image information, the gray-level co-occurrence matrix (GLCM) algorithm was employed to extract the texture features of camellia oil at the characteristic wavelength in this study. Then, the grade classification model of camellia seed oil was established by fusion of spectral and image information. GLCM is a texture feature extraction method based on sample image information, which can describe the relevant information of sample image gray level in azimuth, adjacent spatial position, and azimuth change range [25].

The definition of GLCM is expressed as the probability $h\;\left(i,\;j,d,f\right)$ that the pixel element with gray level $i\;\left(m,\;n\right)$ leaves a certain distance d and reaches the pixel element with gray level $j\;\left(m+a,n+b\right)$. All estimated values could represent a matrix. Its expression is shown in Equation (1).

(1) $$h(i,j,d,f)=\left[\left(m,n\right)\left(m+a,n+b\right)\int \left(m,n\right)\int \left(m+a,n+b\right)\right]$$

where f is the direction of the GLCM generation, and four directions are taken as follows: 0°, 45°, 90°, and 135°.

Four texture feature parameters, contrast $f_{1}$, energy value $f_{2}$, entropy $f_{3}$, and correlation index $f_{4}$, are used to describe the image extracted by the GLCM. The physical meanings of the four texture feature parameters are as follows:

Contrast $f_{1}$ mainly reflects the probability distribution of each pixel of the GLCM, the degree of local change of image pixels, and the clarity and texture depth of the image. The contrast $f_{1}$ is calculated in Equation (2).

(2) $$f_{1}=\sum_{i}\sum_{j}{\left(i,\;j\right)}^{2}h(i,\;j)$$

Energy value $f_{2}$ is used to describe the uniformity of image texture distribution, and the energy value is calculated in Equation (3).

(3) $$f_{2}=\sum_{i}\sum_{j}h^{2}\left(i,\;j\right)$$

Entropy $f_{3}$ is used to describe the image size, and it is calculated in Equation (4).

(4) $$f_{3}=-\sum_{i}\sum_{j}h\left(i,\;j\right)\mathrm{lg}p\left(i,\;j\right)$$

The correlation index $f_{4}$ is used to describe the similar degree of data information in a row or a column direction of the GLCM, which is used as the linear correlation coefficient value of the matrix gray level. The correlation index $f_{4}$ is calculated in Equation (5).

(5) $$f_{4}=\frac{\sum_{i}\sum_{j}\left[{\left(i,j\;\right)}^{2}h(i,j)-\beta_{x}\beta_{y}\;\;\right]}{\alpha_{x}\alpha_{y}}$$

where $\alpha_{x},\alpha_{y}$ are the gray value, and $\beta_{x},\;\beta_{y}$ are the standard deviation.

### 2.5. Model Establishment and Evaluation

**Figure 3:**
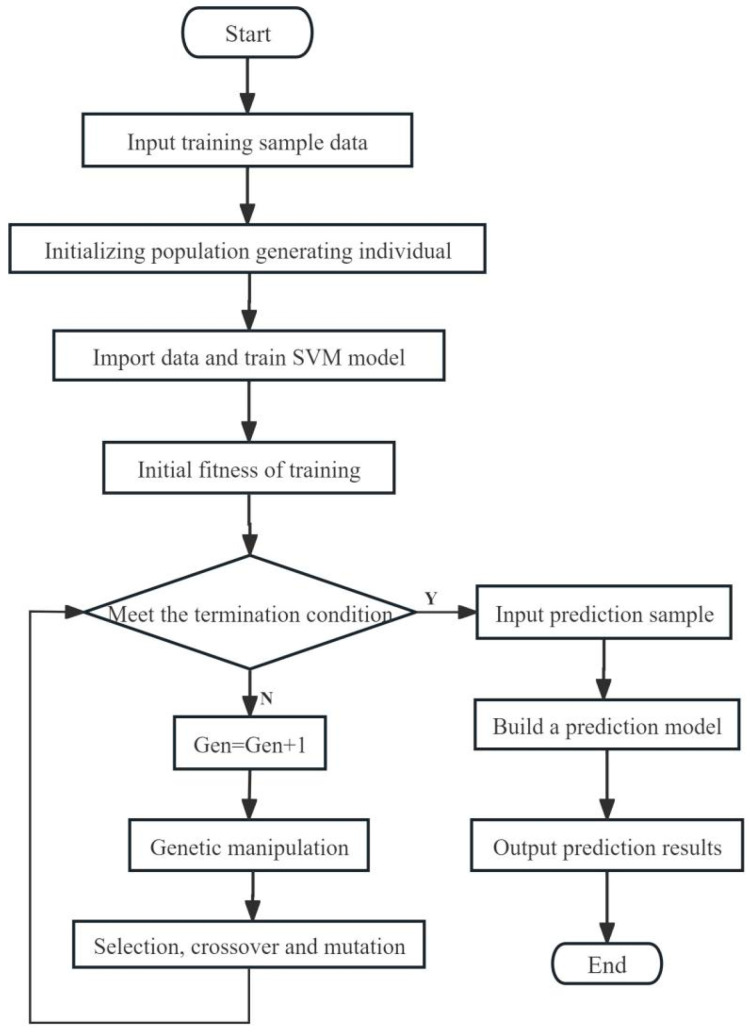
Flow chart of SVM optimized by GA for the grade classification model of camellia seed oil.

The grade classification models for camellia seed oil were developed using a support vector machine (SVM) enhanced by a genetic algorithm (GA) [26]. The support vector machine (SVM) realizes data classification by projecting the data into a high-dimensional data space using the projection relation defined by kernel function. Kernel function and its corresponding kernel function parameter g and penalty factor c are three optional optimization items of SVM. Genetic algorithm (GA) is used to optimize kernel function parameter g and penalty factor c. Genetic algorithm GA seeks the optimal solution by simulating the Darwinian genetic mechanism and natural selection biological evolution mechanism. First, the number of individuals in the first generation is set, then the process of SVM is optimized by evolving the optimal solution, and, finally, the GA-SVM grade classification model of camellia seed oil is established. The establishment process of the grade classification model of camellia seed oil is shown in Figure 3.

In this study, the accuracy of the experiment set was selected to evaluate the performance of the model. Accuracy means the ratio of correctly classifying the number of samples from the total number of samples. The higher the ratio, the better the classification accuracy of the model. The accuracy is calculated in Equation (6).

(6) $$Accuracy=(m_{1}/m_{2})\times 100\%$$

where $m_{1}$ is the correctly classified number of samples, and $m_{2}$ is the total number of samples.

## 3. Results and Discussion

### 3.1. Hyperspectral Analysis of Camellia Seed Oil

The average spectral curves of three different grades of camellia seed oil samples are shown in Figure S2. Generally speaking, the overall trend of the spectra of the three different-grade camellia seed oils was similar. In the range of 870–1200 nm, two distinct strong absorption peaks were observed at 960 nm and 1075 nm. In the range of 1200–1720 nm, two additional strong absorption peaks were detected at 1320 nm and 1585 nm, along with a weak absorption peak at 1510 nm. The characteristic peak and its location related to camellia seed oil matched those found in studies on detecting adulteration levels in camellia seed oil [2,27]. However, the spectra of the three different grade camellia seed oils were clearly distinguished at absorption peaks, and the spectral reflectance of the Grade 3 camellia seed oil was always higher than that of the other two grades in the whole wavelength range. It could be seen that the difference in spectral reflectance indicated that it was feasible to distinguish the grades of camellia seed oil by the spectrum.

### 3.2. Principal Component Analysis for Grade Classification of Camellia Seed Oil (PCA)

**Figure 4:**
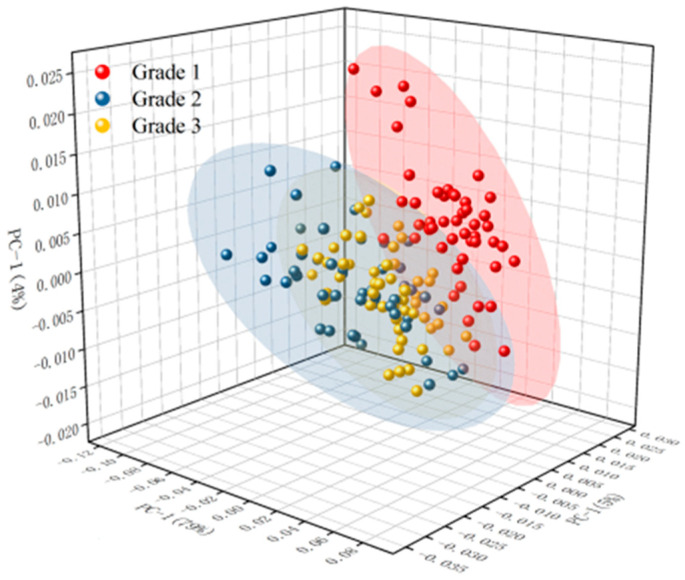
The grade classification results of PCA for camellia seed oil after SNV preprocessing.

The grade classification results of the PCA are shown in Figure 4 and Figure S3. It could be seen from Figure S3a that there was some data clustering in the original spectrum; however, three different grades of camellia oil were cross-mixed together, and the classification characteristics of the original spectrum were not significant. It could be seen from Figure S3b,d,e that the classification effect of the model after SG, 1Der, and 2Der preprocessing was worse than that of the original spectrum, possibly due to the removal of some effective information in the spectrum during preprocessing. It could be seen from Figure 4 and Figure S3c that the model’s classification performance improved after normalization and SNV preprocessing compared to the original spectrum, and the three grades of camellia seed oil could be distinguished basically. Among them, the classification effect of the PCA after SNV preprocessing was optimal. The separability of Grade 1 and Grade 3 samples was obvious, and there was a small amount of cross-mixed samples between the Grade 2 samples and the Grade 1 or Grade 3 samples. A similar conclusion was obtained in the origin identification of dried tangerine peel by near infrared spectroscopy. The identification accuracy of dried tangerine peel origin was 88.60% after SNV preprocessing and a principal component analysis [28]. This finding suggested that SNV preprocessing is an effective method for classification research. For the grade classification of camellia seed oil, the results of PCA after SNV preprocessing showed that there was still a small amount of cross-mixed samples between Grade 2 and Grade 1 or Grade 3 camellia seed oil. It was necessary to continue to optimize the modeling.

### 3.3. Analysis of Grade Classification Model of Camellia Seed Oil Based on Full Wavelength

The grade classification modeling results of camellia seed oil based on full wavelength are shown in Table 3. Based on the original spectrum, the GA-SVM grade classification model for camellia seed oil achieved accuracies of 71.71% for the calibration set and 70.10% for the prediction set, respectively. After 1Der preprocessing, the GA-SVM grade classification model achieved accuracies of 73.00% for the calibration set and 71.33% for the prediction set, respectively. Compared with the model based on the original spectrum, the accuracy of the model established after 1Der preprocessing improved slightly, while the accuracy of the model established after 2Der preprocessing decreased. After 2Der preprocessing, the model’s accuracy for the calibration set and prediction set decreased by 6.98% and 10.00%, respectively. This was consistent with the results of Section 2.2, which may be caused by the low dimension of data and the information loss of dimension reduction. The accuracy of the model established after normalization and SNV preprocessing was significantly improved, which was attributed to the fact that these two preprocessing methods reduced the influence of irrelevant variables and retained the really valuable information in the spectrum. Among them, the model after SNV preprocessing was optimal, which was consistent with the results of the PCA analysis in Section 2.2. The grade classification model for camellia seed oil achieved accuracies of 83.11% for the calibration set and 81.77% for the prediction set, respectively. Compared with the model based on the original spectrum, after SNV preprocessing, the model’s accuracy for the calibration set and prediction set improved by 11.41% and 11.67%, respectively.

### 3.4. Analysis of the Grade Classification Model of Camellia Seed Oil Based on Characteristic Wavelength

In terms of SPA characteristic wavelength selecting, 17 characteristic wavelengths (as shown in Figure S4) were selected, and they were 990.76 nm, 1134.80 nm, 1178.99 nm, 1209.66 nm, 1367.89 nm, 1299.01 nm, 1300.44 nm, 1309.41 nm, 1345.77 nm, 1409.88 nm, 1434.00 nm, 1456.23 nm, 1475.43 nm, 1500.99 nm, 1500.88 nm, and 1567.12 nm, respectively. The characteristic wavelength selected by the SPA method accounted for 11.11% of the full wavelength. Based on characteristic wavelength, the grade classification model (SPA-GA-SVM) of camellia seed oil would be established.

In terms of CARS characteristic wavelength selecting, eight characteristic wavelengths (as shown in Figure S5) were selected, and they were 1137.80 nm, 1278.78 nm, 1312.77 nm, 1367.98 nm, 1488.66 nm, 1598.12 nm, 1677.98 nm, and 1698.12 nm, respectively. The characteristic wavelength selected by the CARS method accounted for 5.22% of the full wavelength. Based on characteristic wavelength, the grade classification model (CARS-GA-SVM) of camellia seed oil would be established.

Table 4 presents the results of the characteristic wavelength selection by SPA and CARS. The wavelengths selected by SPA accounted for 11.11% of the full spectrum, while those selected by CARS accounted for 5.22%. It could be seen that the amount of data was greatly reduced after the characteristic wavelength selection, which reduced the amount of data calculation and improved the calculation efficiency.

The results of the grade classification model of camellia seed oil based on the characteristic wavelength are shown in Table 5. The accuracy of the calibration set and prediction set of the SPA-GA-SVM model were 85.01% and 80.56%, respectively, and those of the CARS-GA-SVM model were 88.76% and 86.23%, respectively. Compared with those of the model based on full wavelength, the accuracy of the calibration set and the prediction set of the CARS-GA-SVM model were improved by 18.28% and 18.15%, respectively. It can be concluded that some redundant wavelengths were eliminated, which made the characteristic wavelength information more accurate to distinguish different grades of camellia seed oil. The characteristic wavelength selection was an effective method to improve the accuracy of the classification model. In the aspect of characteristic wavelength modeling, the CARS-GA-SVM model was optimal. Similar results were also found in the prediction of soluble solids content (SSC) in apples by hyperspectral coupled with wavelength selection. CARS could effectively extract the characteristics of SSC. The prediction model based on the CARS-SPA wavelength selection algorithm shows great potential for the online detection of apple SSC [29]. In the study of wavelength variable selection methods for the prediction of SOM (soil organic matter) by hyperspectral image technology, Yu et al. (2016) found that the CARS-SPA method selected 37 characteristic wavelengths from the 2001 full wavelength, and the selected wavelength only accounted for 1.85% of the full wavelength. The CARS-SPA-PLSR model was rather simple, had a good prediction effect, and could be used as an important method to estimate SOM [30].

**Figure 5:**
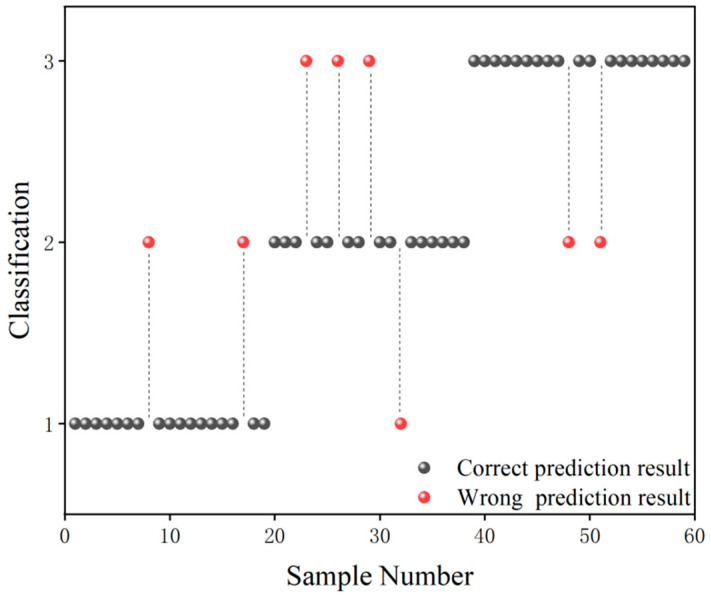
Grade classification prediction result of the CARS-GA-SVM model.

The grade classification prediction result of the CARS-GA-SVM model is shown in Figure 5, in which the black ball indicates the correct prediction result and the red ball indicates the wrong prediction result. As can be seen from Figure 5, in terms of Grade 1 samples, only two Grade 1 samples were misclassified as Grade 2 samples. In terms of Grade 2 samples, only one Grade 2 sample was misclassified as a Grade 1 sample, and three Grade 2 samples were misclassified as Grade 3 samples. In terms of Grade 3 samples, only two were misclassified as Grade 2 samples. In general, the grade classification prediction ability of the CARS-GA-SVM model was high.

### 3.5. Analysis of the Grade Classification Model of Camellia Seed Oil Fusing Spectral and Image Features

According to the results in Section 3.4, eight characteristic wavelengths were selected by the CARS method. In the following study, combined with spectral information and hyperspectral image information under characteristic wavelength, the grade classification model of camellia seed oil fusing spectral and image features would be established. Figure S6 showed the hyperspectral images of sample 98 of camellia seed oil at eight characteristic wavelengths. As shown in Figure S6, the contrast $f_{1}$, energy value $f_{2}$, entropy $f_{3}$, and correlation index $f_{4}$ of each image were variant at different characteristic wavelengths. The GLCM algorithm was used to extract image features from characteristic wavelength images, and four directions of 0°, 45°, 90°, and 135° with a total of 16 parameters were extracted in this study. In this study, a circular mask image was employed to cover the background so as to make the ROI of camellia seed oil. In order to extract effective information in the image, the PCA method was used to analyze the principal component of the image, and the first, second, and third principal component images of the GLCM texture image were obtained, which were used for image texture feature extraction.

The results of the grade classification model of camellia seed oil fusing spectral and image features (CARS-GLCM-GA-SVM) are shown in Table 6. It can be seen from the Table that the accuracy of the calibration set and the prediction set of the CARS-GLCM-GA-SVM model established without a PCA analysis were 90.33% and 87.33%, respectively, which was not significantly improved compared with those of the CARS-GA-SVM model (88.76% and 86.23%, respectively) based on characteristic wavelength. After an image PCA analysis, the accuracy of the calibration set and the prediction set of the CARS-GLCM-GA-SVM model significantly improved. The CARS-GLCM-GA-SVM model based on the PC3 was optimal, and its accuracy of the calibration set and the prediction set were 98.30% and 96.61%, respectively. Compared with those of the CARS-GA-SVM model, the accuracy of the calibration set and the prediction set were improved by 9.54% and 10.38%, respectively. In research on determining the damage degree of yellow peaches based on hyperspectral image fusion technology, Li et al. (2023) found that the model established by combining characteristic wavelength with image features had the best classification prediction effect, and the prediction accuracies of lightly bruised, moderately bruised, and heavily bruised yellow peaches were 95%, 90%, and 95%, respectively [31]. Similarly, in the study of fast nondestructive discrimination of black tea grade, compared with the traditional GA-SVM model, the prediction accuracy of the model established by combining characteristic wavelength with image features was improved from 78.33% to 96.67% [32]. These studies show that fusing spectral and image features significantly improved the prediction accuracy of the established model.

**Figure 6:**
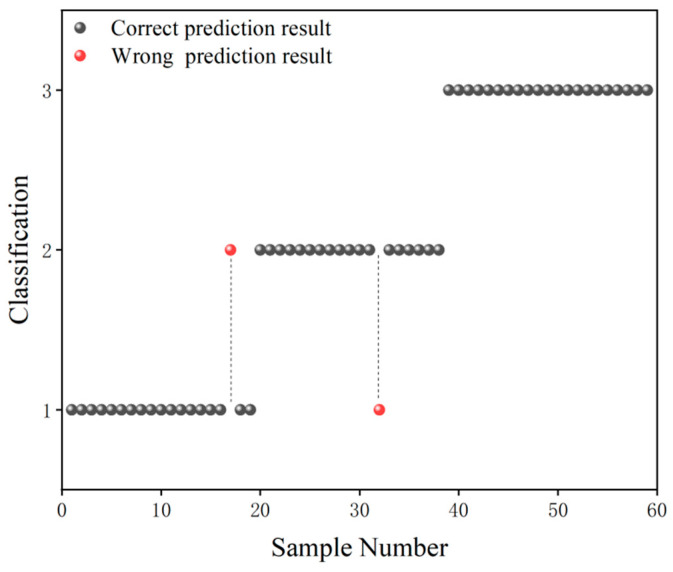
Grade classification prediction result of the CARS-GLCM-GA-SVM model.

The grade classification prediction result of the CARS-GLCM-GA-SVM model is shown in Figure 6, in which the black ball indicates the correct prediction result and the red ball indicates the wrong prediction result. As can be seen from Figure 6, in terms of Grade 1 samples, only one Grade 1 sample was misclassified as a Grade 2 sample. In terms of Grade 2 samples, only one Grade 2 sample was misclassified as a Grade 1 sample. In terms of Grade 3 samples, there were no cross-mixed samples with the Grade 1 or Grade 2 samples. According to the results in Figure 6, the prediction accuracy of Grade 3 camellia seed oil was 100%, and the prediction accuracy of Grade 1/2 camellia seed oil was higher than 95%.

## 4. Conclusions

Hyperspectral imaging technology was utilized to investigate the grade classification of three distinct grades of camellia seed oil. Based on the genetic algorithm (GA) and support vector machine (SVM), the optimal grade classification based on full wavelength, characteristic wavelength, and fusing spectral and image features was established successively. The GA-SVM model based on full wavelength achieved 83.11% accuracy for the calibration set and 81.77% for the prediction set. Compared with the model based on the original spectrum, the accuracy of the calibration set and the prediction set of the model established after SNV preprocessing were improved by 11.41% and 11.67%, respectively. For the optimal grade classification model (CARS-GA-SVM) based on characteristic wavelength, the accuracy of the calibration set and the prediction set of the CARS-GA-SVM model were 88.76% and 86.23%, respectively. Compared with those of the GA-SVM model based on full wavelength, the accuracy of the calibration set and the prediction set of the CARS-GA-SVM model were further improved by 5.65% and 4.46%, respectively. For the optimal grade classification model (CARS-GLCM-GA-SVM) fusing spectral and image features, the accuracy of the calibration set and the prediction set of the CARS-GLCM-GA-SVM model were 98.30% and 96.61%, respectively. Compared to the CARS-GA-SVM model, the CARS-GLCM-GA-SVM model improved the calibration accuracy by 10.75% and the prediction accuracy by 12.04%, respectively. Compared to the GA-SVM model, the CARS-GLCM-GA-SVM model improved the calibration accuracy by 18.28% and the prediction accuracy by 18.15%, respectively. This study demonstrates that hyperspectral imaging is a viable and promising technique for classifying and identifying edible oils.

## Figures and Tables

**Table 1:** The sensory evaluation criteria of different grades of camellia seed oil.

Grade	Color	Smell	Taste	Touch
Grade 1	Bright and golden yellow	Rich, mellow	Smooth, obvious fragrance	Moist but not greasy
Grade 2	Slightly bright and light yellow	Slightly rich, slightly mellow	Slightly smooth, slightly fragrant	Slightly moist but not greasy
Grade 3	Opaque, turbid, and dark yellow	Not fragrant and smelly	Poor, obvious astringency	Greasy

**Table 2:** Camellia seed oil sample division results.

Grade of Camellia Seed Oil	Samples/Number	Calibration Set/Number	Prediction Set/Number
Grade 1	48	29	19
Grade 2	49	31	18
Grade 3	56	32	24
Total	153	92	61

**Table 3:** Grade classification models of camellia seed oil based on full wavelength.

Preprocessing Method	Penalty Factor	Kernel Function Parameter	Accuracy of Calibration Set/%	Accuracy of Prediction Set/%
Origin	5.53	6.91	71.71	70.10
SG	6.23	6.52	72.77	69.91
1Der	6.21	6.32	73.00	71.33
2Der	6.99	7.22	64.72	60.10
Normalize	5.49	6.07	80.32	79.44
SNV	5.19	5.35	83.11	81.77

**Table 4:** The results of characteristic wavelength selection by SPA and CARS.

Method	Wavelength/Number	Characteristic Wavelengths/nm	Proportion of Full Wavelength/%
SPA	17	990.76, 1134.80, 1178.99, 1209.66, 1267.89, 1299.01, 1300.44, 1309.41, 1345.77,1367.89, 1409.88, 1434.00, 1456.23, 1475.43, 1500.99, 1500.88, 1567.12	11.11%
CARS	8	1137.80, 1278.78, 1312.77, 1367.98, 1488.66, 1598.12, 1677.98, 1698.12	5.22%

**Table 5:** Grade classification models of camellia seed oil based on characteristic wavelength.

Method	Penalty Factor	Kernel Function Parameter	Accuracy of Calibration Set/%	Accuracy of Prediction Set/%
SPA	5.40	6.22	85.01	80.56
CARS	5.03	6.34	88.76	86.23

**Table 6:** Grade classification models of camellia seed oil fusing spectral and image features.

Method	Penalty Factor	Kernel Function Parameter	Accuracy of Calibration Set/%	Accuracy of Prediction Set/%
None	5.68	4.03	90.33	87.33
PC1	5.48	6.78	92.22	90.66
PC2	4.80	4.71	94.65	91.08
PC3	4.01	1.12	98.30	96.61

## Data Availability

The original contributions presented in the study are included in the article/Supplementary Materials, further inquiries can be directed to the corresponding author.

## Supplementary Materials

The following supporting information can be downloaded at: https://www.mdpi.com/article/10.3390/foods13203331/s1, Figure S1. Three different grades of camellia seed oils ((a) Grade 1, (b) Grade 2, and (c) Grade 3; Figure S2. The typical average hyper-spectrum of different grades of camellia seed oil samples; Figure S3. The grade classification results of PCA for camellia seed oil after (a) original spectrum, (b) SG, (c) Normalize, (d) 1Der, (e) 2Der; Figure S4. Characteristic wavelength of camellia seed oil selected by SPA method; Figure S5. Characteristic wavelength of camellia seed oil selected by CARS method: (a) Variation trend of the number of variables with the number of samples; (b) RMSECV; (c) The change process of regres-sion coefficient of each variable with sampling times (The blue line represents the position with the lowest RMSECV); Figure S6. Hyperspectral image at characteristic wavelength of camellia seed oil (Sample 98).
