# Supplementary material for: Grade Classification of Camellia Seed Oil Based on Hyperspectral Imaging Technology
Source: Foods. 2024 Oct 20;13(20):3331. doi: 10.3390/foods13203331 (PMC11507928; doi:10.3390/foods13203331)
Supplement: Supplementary file 1 [file foods-13-03331-s001.zip › foods-3238092-supplementary.pdf]

Supplementary Materials For

Grade Classification of Camellia Seed Oil Based on Hyperspectral Imaging Technology

Yuqi Gu [1], Jianhua Wu [2], Yijun Guo [1], Sheng Hu [1], Kaixuan Li [3], Yuqian Shang [4], Liwei Bao [1], Muhammad Hassan [5] and Chao Zhao [1,*]

[1] College of Optical, Mechanical and Electrical Engineering, Zhejiang A&F University, Hangzhou 311300, China
[2] Panzhihua Academy of Agriculture and Forestry Sciences, Panzhihua 617061, China
[3] National Engineering Technology Research Center of Forestry and Grassland Machinery for Hilly and Mountainous Areas, State Forestry and Grassland Administration, Hangzhou 311300, China
[4] Key Laboratory of Agricultural Equipment for Hilly and Mountainous Areas in Southeastern China, Ministry of Agriculture and Rural Affairs, Hangzhou 311300, China
[5] U.S.-Pakistan Center for Advanced Studies in Energy, National University of Sciences and Technology (NUST), Islamabad 44000, Pakistan
* Correspondence: zhaochao@zafu.edu.cn

Supplementary Figure Captions

Figure S1. Three different grades of camellia seed oils ((a) Grade 1, (b) Grade 2, and (c) Grade 3

Figure S2. The typical average hyper-spectrum of different grades of camellia seed oil samples

Figure S3. The grade classification results of PCA for camellia seed oil after (a) original spectrum, (b) SG, (c) Normalize, (d) 1Der, (e) 2Der

Figure S4. Characteristic wavelength of camellia seed oil selected by SPA method

Figure S5. Characteristic wavelength of camellia seed oil selected by CARS method: (a) Variation trend of the number of variables with the number of samples; (b) RMSECV; (c) The change process of regres-sion coefficient of each variable with sampling times (The blue line represents the position with the lowest RMSECV)

Figure S6. Hyperspectral image at characteristic wavelength of camellia seed oil (Sample 98)

Figure S1. Three different grades of camellia seed oils ((a) Grade 1, (b) Grade 2, and (c) Grade 3

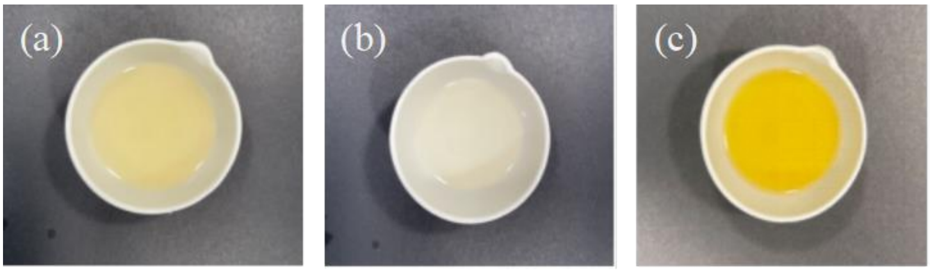

Figure S2. The typical average hyper-spectrum of different grades of camellia seed oil samples

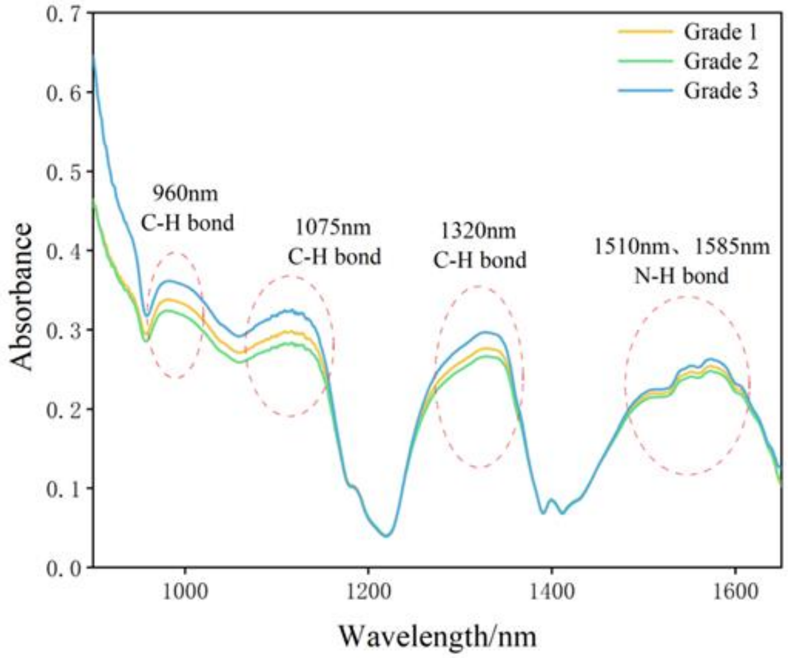

Figure S3. The grade classification results of PCA for camellia seed oil after (a) original spectrum, (b) SG, (c) Normalize, (d) 1Der, (e) 2Der

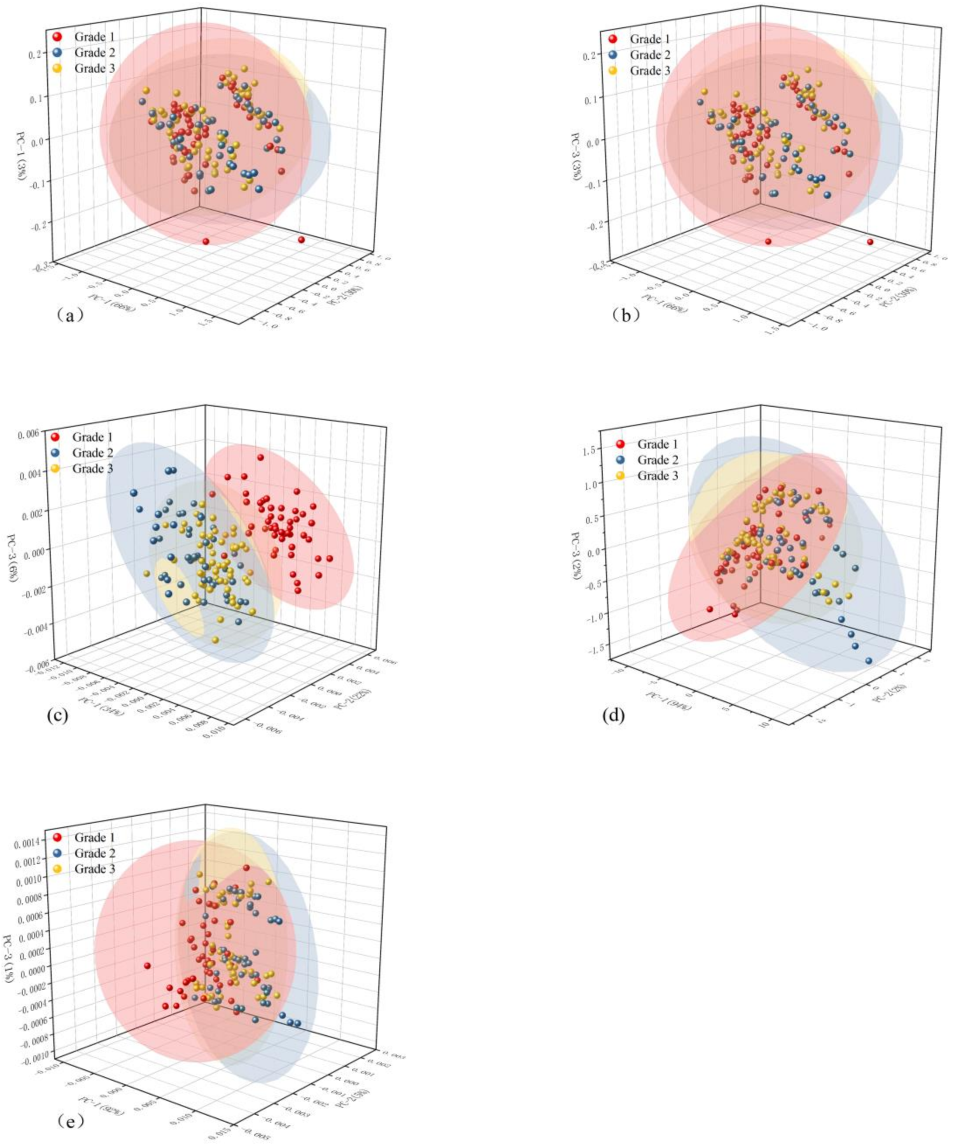

Figure S4. Characteristic wavelength of camellia seed oil selected by SPA method

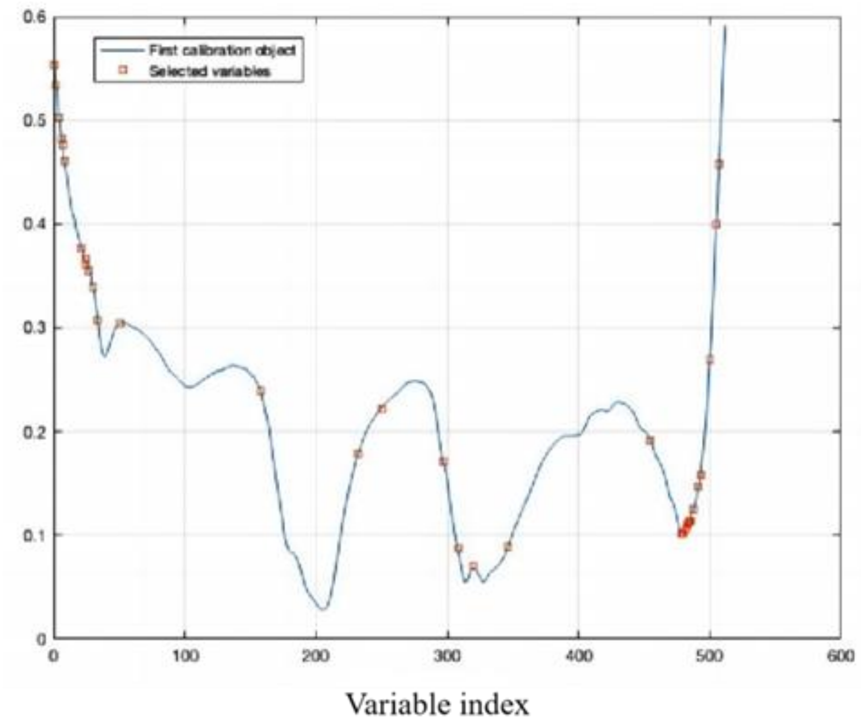

Figure S5. Characteristic wavelength of camellia seed oil selected by CARS method: (a) Variation trend of the number of variables with the number of samples; (b) RMSECV; (c) The change process of regres-sion coefficient of each variable with sampling times (The blue line represents the position with the lowest RMSECV)

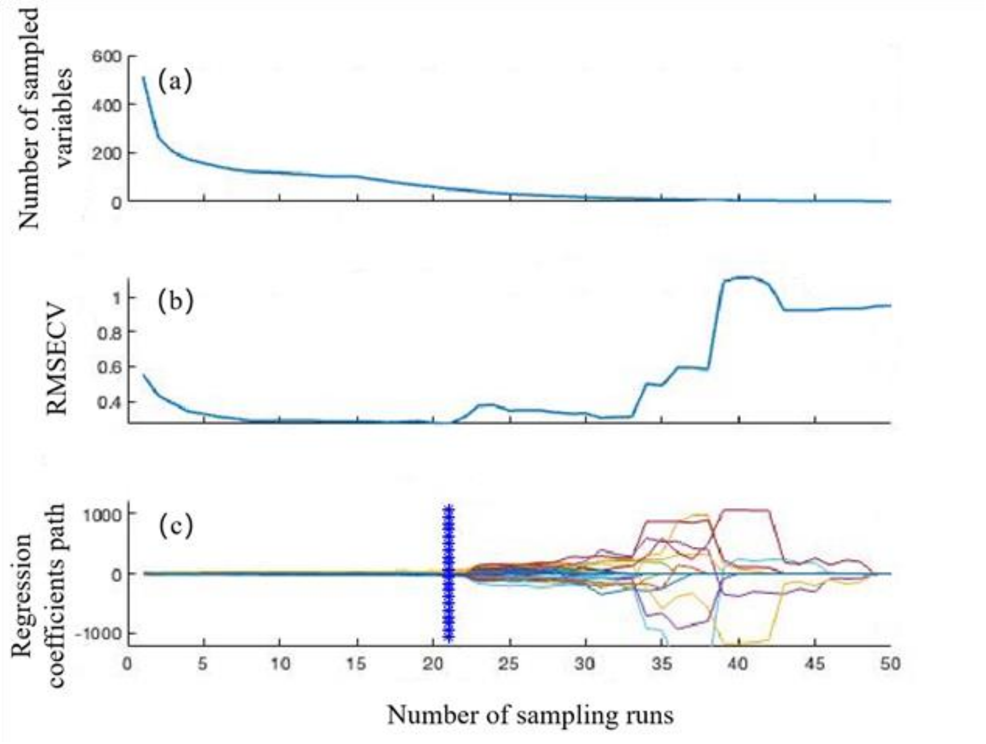

Figure S6. Hyperspectral image at characteristic wavelength of camellia seed oil (Sample 98)

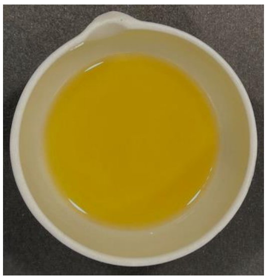

(**a**) 1137.80nm

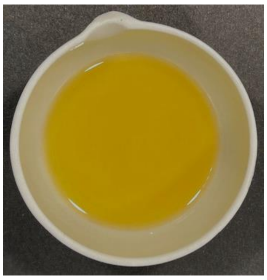

(**b**) 1278.78nm

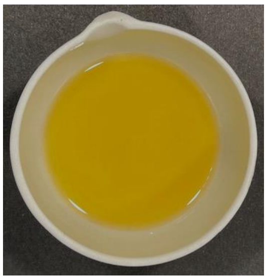

(**c**) 1312.77nm

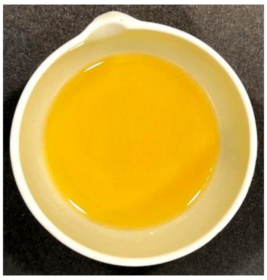

(**d**) 1367.98nm

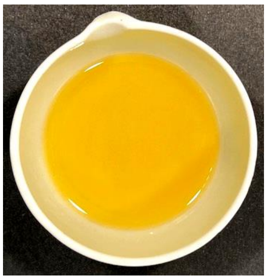

(**e**) 1488.66nm

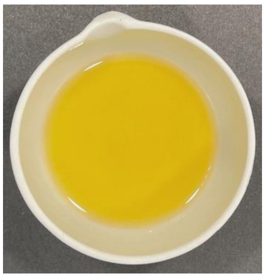

(**f**) 1598.12nm

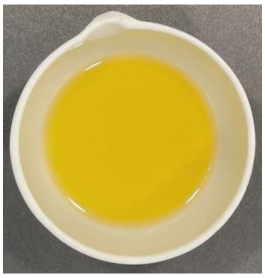

(**g**) 1677.98nm

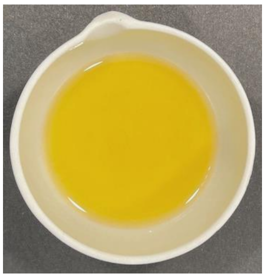

(**h**) 1698.12nm
